# Oral microbiota transplantation for intra-oral halitosis: a feasibility analysis based on an oral microbiota colonization trial in Wistar rats

**DOI:** 10.1186/s12866-024-03322-4

**Published:** 2024-05-17

**Authors:** Zhiqiang Huang, Yongbo Cheng

**Affiliations:** Department of Gastroenterology I, The First Affiliated Hospital of Xinjiang Medical University, 393th Xinyi Road, Urumqi, Xinjiang 830054 China

**Keywords:** Intra-oral halitosis, Oral flora, Flora imbalance, Microbiota transplantation, 16SrRNA gene sequencing, PICRUSt metabolic analysis

## Abstract

**Background:**

Intra-oral halitosis (IOH) is bad breath produced locally by the mouth in addition to systemic diseases and is one of the main causes of interpersonal communication and psychological disorders in modern society. However, current treatment modalities still only alleviate IOH and do not eradicate it. Therefore, based on the differential performance of oral microecology in IOH patients, we propose a microbiota transplantation treatment aimed at restoring oral microecological balance and analyze its feasibility by oral flora colonization test in Wistar rats.

**Objective:**

Saliva flora samples were collected from IOH patients and healthy subjects to analyze the feasibility of oral microbiota transplantation (OMT) for the treatment of IOH by the Wistar rat oral flora colonization test.

**Methods:**

Seven patients with IOH who visited the First Affiliated Hospital of Xinjiang Medical University from June 2017 to June 2022 with the main complaint of halitosis and three healthy subjects were randomly selected. A Halimeter portable breath detector was used to record breath values and collect saliva flora samples. Sixteen SPF-grade male Wistar rats were housed in the Animal Experiment Center of Xinjiang Medical University and randomly divided into an experimental group (Group E) and a control group (Group C) for the oral flora colonization test. Species composition and associated metabolic analysis of oral flora during the Wistar rat test using 16SrRNA sequencing technology and PICRUSt metabolic analysis. Also, the changes in the breath values of the rats were recorded during the test.

**Results:**

The proportion of *Porphyromonas*, *Fusobacterium*, *Leptotrichia*, and *Peptostreptococcus* was significantly higher in group E compared to group C after colonization of salivary flora of IOH patients (all *P* < 0.05), and the abundance with *Gemella* was zero before colonization, while no colonization was seen in group C after colonization compared to baseline. PICRUSt metabolic analysis also showed significantly enhanced IOH-related metabolic pathways after colonization in group E (all *P* < 0.05), as well as significantly higher breath values compared to baseline and group C (all *P* < 0.0001). After colonization by salivary flora from healthy subjects, group E rats showed a decrease in the abundance of associated odor-causing bacteria colonization, a reduction in associated metabolism, and a significant decrease in breath values. In contrast, group C also showed differential changes in flora structure and breath values compared to baseline after salivary flora colonization of IOH patients.

**Conclusions:**

OMT for IOH is a promising green treatment option, but the influence of environmental factors and individual differences still cannot be ignored.

## Background

Intra-oral halitosis (IOH) is a common disorder of oral flora dysbiosis, the long-term recurrence of the symptoms brings unpleasant feelings, which leads to IOH patients showing fear of communication, anxiety, depression, low self-esteem, distress, and other negative emotions, serious affecting people’s social interactions and physical and mental health [1–4]. Epidemiological studies have shown that the prevalence of halitosis in China is about 27.5%, while in Western countries it can be as high as 50%, with IOH accounting for more than 85% of the cases [5]. Previously, the causes of IOH were attributed to some oral diseases (periodontitis and gingivitis, etc.) and it has been ranked as the third most common reason for dentistry visits (the first two being caries and periodontal disease, respectively) [6], but in recent years there has been an increase in the number of patients presenting to gastroenterology clinics with complaints of halitosis, the vast majority of whom report short-term effectiveness after professional oral interventions (scaling and scraping, etc.), but the long-term results are still unsatisfactory. However, no clear systemic disease was found after a gastroenterological examination. The causes of IOH are still not fully understood and are influenced by various factors such as oral microecological environment, dietary habits, hygiene habits [7], living environment, autoimmunity, and drugs. As the quality of life continues to improve, the number of people with halitosis treatment needs is also increasing, but at this stage, the treatment of IOH is still limited to antibacterial mouthwash, tongue cleaning, dental care, and the use of some antibiotics, which can only alleviate IOH and do not achieve the purpose of eradication. It is undoubtedly a new direction to further explore greener and more effective targeted treatment for the characteristics of oral flora structure of IOH patients [8].

The microbiota in the human ecological environment is known as a major vital organ of the body, it is a key component of the mucosal barrier function, innate and adaptive immune response, and it also acts as an inhibitor of pathogen colonization, which will cause adverse consequences if the flora is disturbed [9]. The digestive tract has different ecological sites for bacterial survival and therefore carries the largest bacterial density in the human body [10]; and the oral cavity, as the beginning of the digestive tract, has suitable conditions for microbial survival because it is connected to the external environment and therefore becomes a suitable zone for the growth of microbial diversity. To date, 29 phylum and 365 genera of oral microbiota have been identified, of which the most prominent genera are *Streptococcus*, *Gemella*, *Granulicatella*, *Rothia*, *Neisseria*, and *Prevotella* [11]. These highly abundant genera have specific adherents on their surfaces that bind to specific glycoprotein receptors on different ecological sites of the oral cavity and eventually colonize them in a selective manner, constituting resident flora on different surfaces of the oral cavity [12, 13], which often represent the uniqueness of microorganisms in different ecological sites of the oral cavity.

In recent years [14–18], high-throughput sequencing techniques have revealed that *Fusobacterium nucleatum*, *Porphyromonas gingivalis*, *Treponema denticola*, *Solobacterium moorei*, *Prevotella intermedia*, *Tannerella forsythia*, *Peptostreptococcus*, and *nanobacteria* have been closely associated with IOH, and in vitro culture techniques have confirmed that *F.nucleatum*, *P.gingivalis*, and *T.denticola* all break down sulfur-containing substrates (sulfur-containing amino acids such as cysteine, methionine, tryptophan, arginine and lysine [18–20]), which in turn release volatile sulfur compounds (VSCs) to produce odor. However, there are approximately 500–700 species of bacteria in the oral cavity [11, 21], and their gene pool is approximately 100 times more diverse than that of their hosts [22], so research continues to target odor-producing bacteria. It has been shown [23, 24] that there are significant differences in the microbial structure of saliva and tongue in patients with IOH, and oral microbiota transplantation (OMT) therapy, inspired by fecal flora transplantation (FMT) therapy, with the goal of restoring oral flora structure, has shown great promise. In this study, the feasibility of OMT for IOH was further explored in depth through an oral flora colonization test in Wistar rats.

## Results

### Sequencing, quality control, and ASV (amplicon sequence variants)

**Fig. 1:**
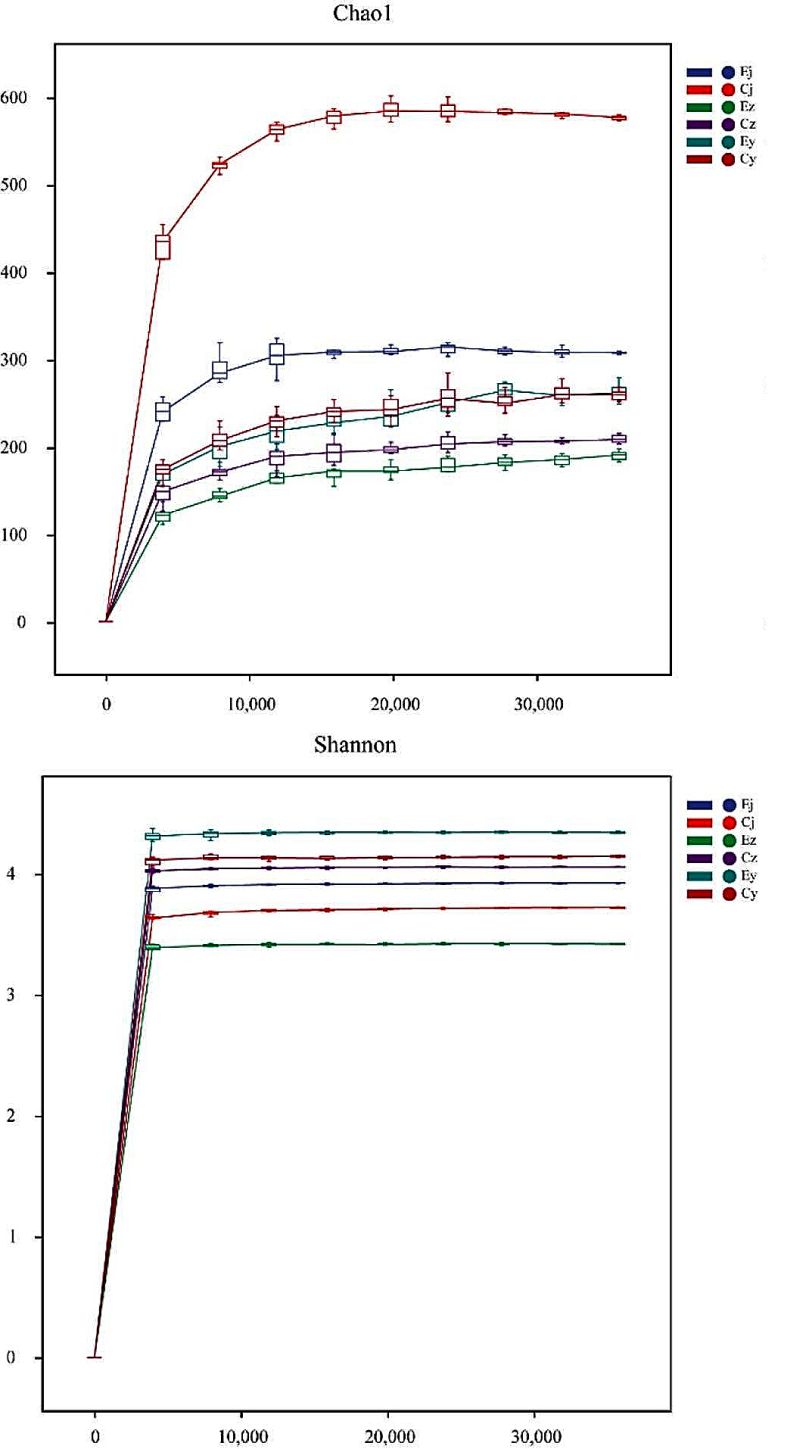
Sparse curve determined by Chao1 index and Shannon index. The flatness of the curve reflects the effect of sequencing depth on sample diversity, the flatter the curve, the more the sequencing result is sufficient to reflect the diversity contained in the sample, and further increasing the sequencing depth can no longer detect a large number of new ASVs that have not yet been found; Ej, Ez, Ey: group E, Cj, Cz, Cy: group C; the letters j, z, and y represent the basal oral flora sample, the post-colonization sample of salivary flora from IOH patients, and the post-colonization sample of salivary flora from healthy subjects, respectively

**Fig. 2:**
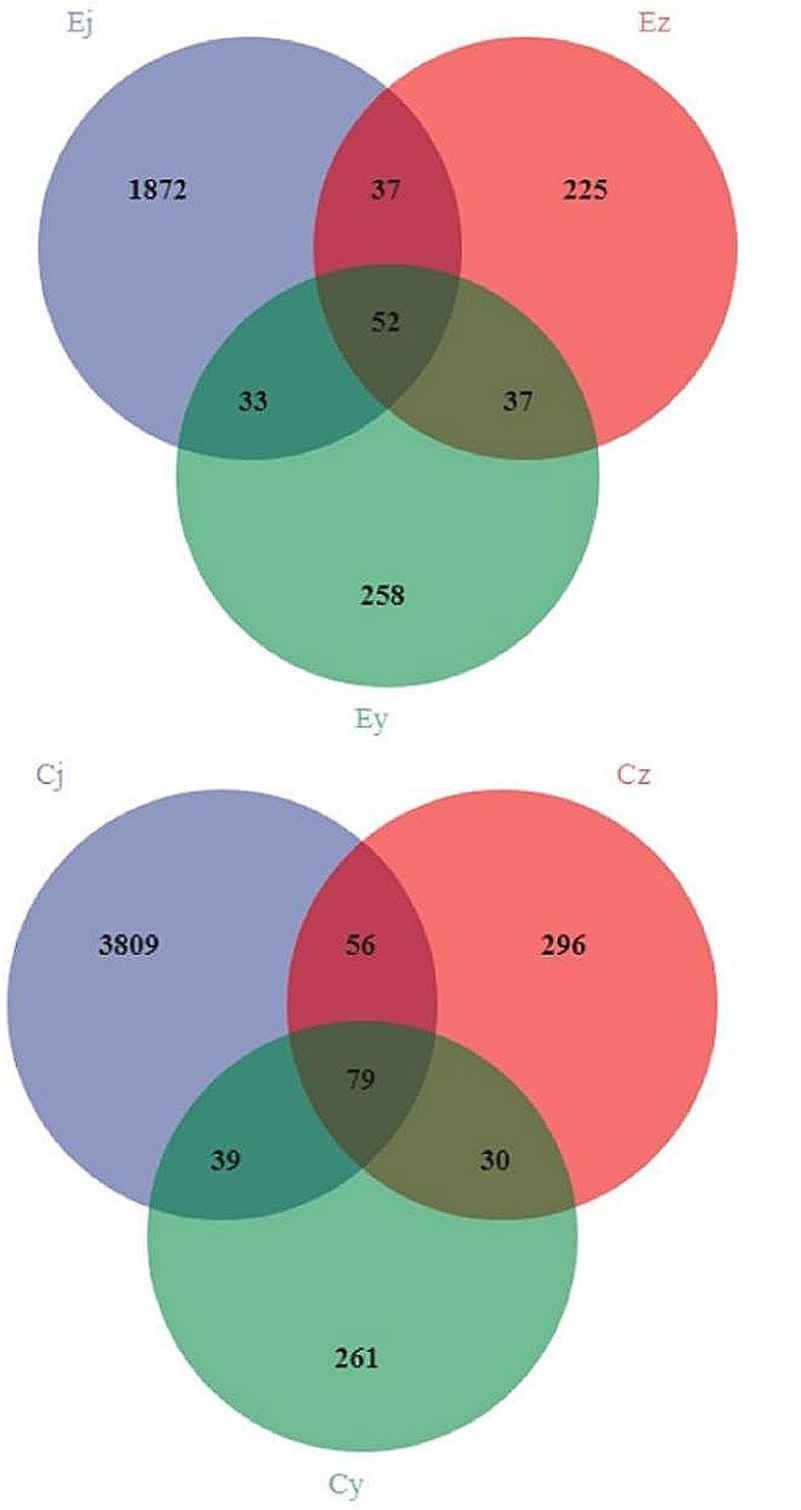
Venn diagram of ASVs of oral flora samples from rats. Ej, Ez, Ey: group E, Cj, Cz, Cy: group C. The letter j represents the rat basal oral flora sample, z represents the post-colonization sample of salivary flora from IOH patients, and y represents the post-colonization sample of salivary flora from healthy subjects

A total of 30 oral flora samples from 16 rats were sequenced, and the average number of downstream data obtained per sample was 100,451. Quality control was performed on the downstream data, and the average number of high-quality data obtained per sample was 76,736, with a quality control efficiency of 76% and an average sequence read length of 421.9 bp. The sparse curves determined by the Shannon index and Chao1 index both tend to be flat, suggesting that the amount of sequencing data and the sequencing depth of all samples are reasonable and can fully reflect the microbial information of the samples (Fig. 1). High-quality de-duplicated sequences of rat oral flora samples were clustered and species annotated at 100% similarity for ASVs, and a total of 7084 ASVs were annotated. In group E, the number of ASVs in the samples of basal oral flora, after salivary flora colonization of IOH patients and after salivary flora colonization of healthy subjects were 1994, 351 and 380, respectively (Fig. 2). And in group C, the number of ASVs in the samples of basal oral flora, after salivary flora colonization of IOH patients and after salivary flora colonization of healthy subjects were 3983, 461 and 409, respectively (Fig. 2).

### Oral flora diversity

**Fig. 3:**
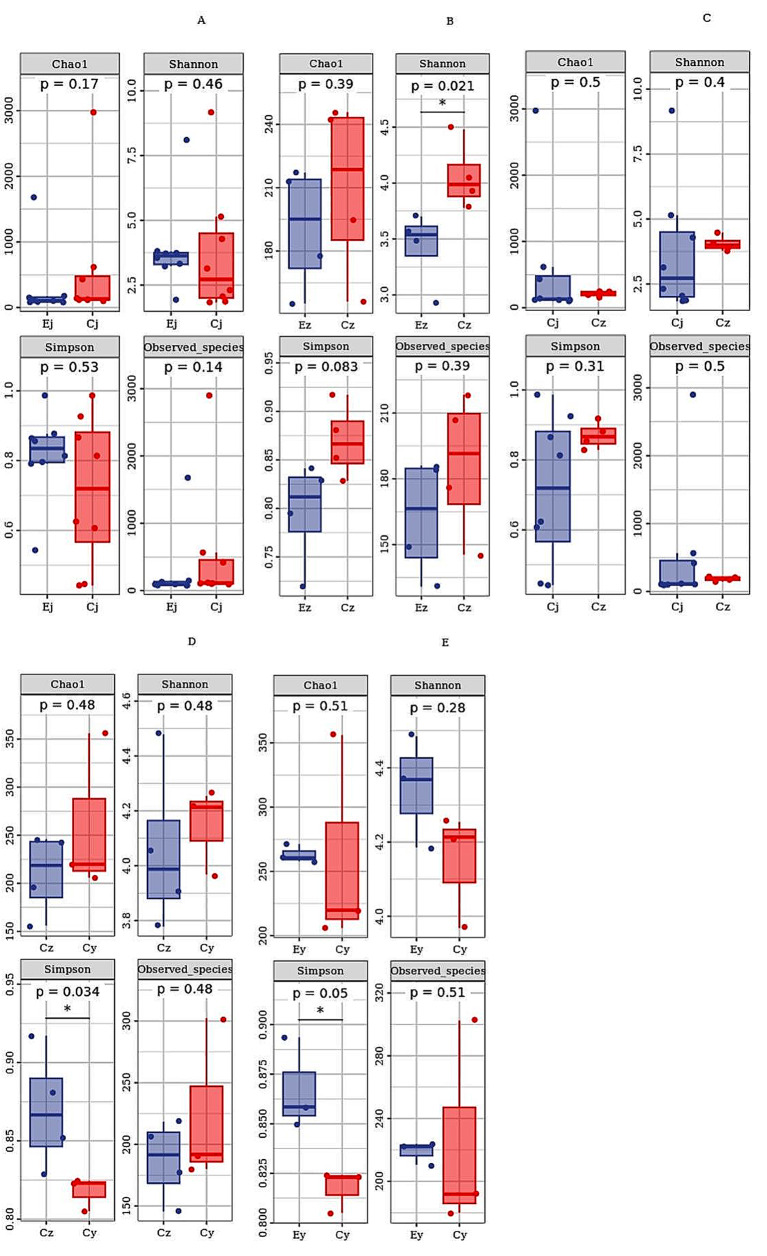
Box plots of alpha diversity of the oral cavity in two groups of rats. Ej, Ez, Ey: group E, Cj, Cz, Cy: group C. The letter j represents the rat basal oral flora sample, z represents the post-colonization sample of salivary flora from IOH patients, and y represents the post-colonization sample of salivary flora from healthy subjects

The α-diversity results showed that no significant differences were seen in the abundance and diversity of the basal oral flora in both groups E and C (all *P* > 0.05) (Fig. 3A). Compared to IOH patients salivary flora colonized in group C, the oral flora diversity was significantly lower in group E rats (Shannon index *P* = 0.021) while the difference in flora abundance was not significant (*P* > 0.05) (Fig. 3B). The differences in abundance and diversity of oral flora before and after salivary flora colonization of IOH patients in group C were not significant (all *P* > 0.05) (Fig. 3C). However, compared to healthy subjects’ salivary flora with no colonization in Group C, oral flora diversity was significantly lower in rats after colonization (Simpson index *P* = 0.034), while there was no significant difference in flora abundance (*P* > 0.05) (Fig. 3D). Compared with the oral flora of healthy subjects in Group C after salivary flora colonization, the oral flora diversity was elevated in Group E rats, while the difference in flora abundance was not significant (*P* > 0.05) (Fig. 3E).

**Fig. 4:**
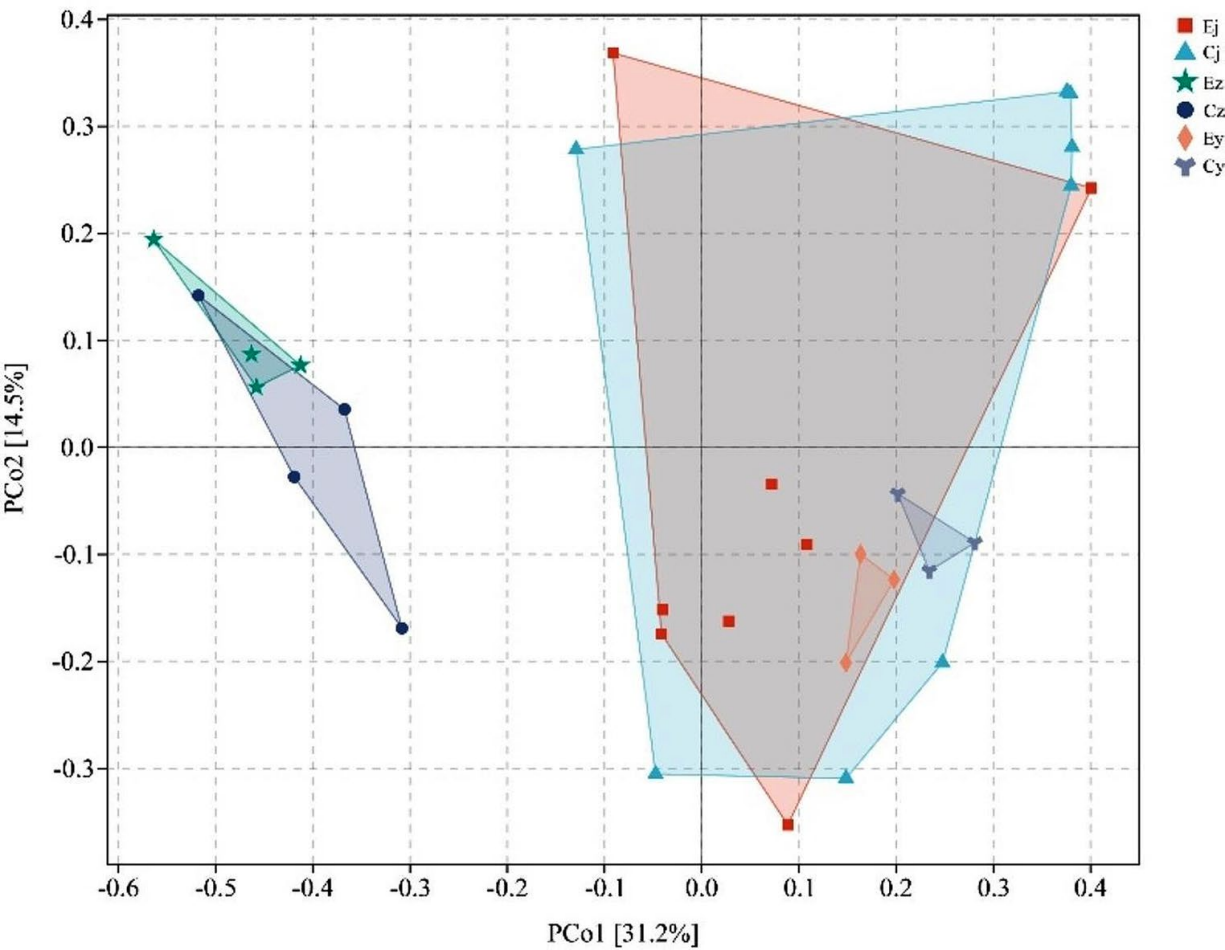
PCoA plots of beta diversity of the rats’ oral cavity. Each point in the figure indicates a flora sample, and the same color indicates the same group; Ej, Ez, Ey: group E, Cj, Cz, Cy: group C. Letter j represents rat basal oral flora sample, z represents IOH patient saliva flora post-colonization sample, and y represents healthy subject saliva flora post-colonization sample

The results of PCoA analysis based on bray_curtis in group E and group C showed that when PCo1 was 31.2% and PCo2 was 14.5%, there was no significant trend of separation between groups of basal oral flora in the two groups of rats, while the salivary flora in group C rats with IOH before and after colonization and the salivary flora with healthy subjects before and after colonization were each aggregated within the group and showed the different location and morphological distribution between groups (Fig. 4). Adonis analysis showed that the difference between the two groups of rats with basal oral flora (R^2^ = 0.080, *P* = 0.261) was not significant. Meanwhile, the oral flora before and after salivary flora colonization in group C rats with IOH (R^2^ = 0.347, *P* = 0.008) and the oral flora before and after salivary flora colonization with healthy subjects (R^2^ = 0.664, *P* = 0.021) components was significantly different. However, the differences between the two groups of rats with IOH after salivary flora colonization (R^2^ = 0.349, *P* = 0.054) and healthy subjects after salivary flora colonization (R^2^ = 0.422, *P* = 0.100) were not significant.

### Analysis of species differences at the phylum and genus level

At the phylum level, only *Cyanobacteria* was significantly increased in the top 50 phyla in abundance among the basic oral flora in groups E and C (*P* = 0.017, Z=-2.381), while the differences in the remaining phyla were not statistically significant (all *P* > 0.05). Compared with Group E before salivary flora colonization of IOH patients, the proportions of *Firmicutes* (Z=-2.717, *P* = 0.007), *Proteobacteria* (Z=-2.038, *P* = 0.042), and *Cyanobacteria* (Z=-2.466, *P* = 0.014) were significantly lower after colonization, while the proportions of *Fusobacteria* (Z=-3.030, *P* = 0.002) and *TM7* (Z=- 2.324, *P* = 0.020) proportions were significantly higher. In contrast, the proportions of *Actinomycetes* (Z=-2.208, *P* = 0.027), *Cyanobacteria* (Z=-2.766, *P* = 0.006), and *TM7* (Z=-1.988, *P* = 0.047) were significantly lower in the oral flora sampled at the same time in group C. The differences between the remaining phyla were not significant (all *P* > 0.05). Compared to healthy subjects’ salivary flora with no colonization in Group E, the proportion of *Firmicutes* (Z=-2.121, *P* = 0.034) was significantly higher after colonization, while the proportion of *Actinomycetes* (Z=-2.121, *P* = 0.034) was significantly lower. In contrast, compared to healthy subjects’ salivary flora with no colonization in Group C, the *Actinobacteria* (Z=-2.121, *P* = 0.034) and *Fusobacteria* (Z=-2.341, *P* = 0.019) increased significantly and the *Proteobacteria* (Z=-2.121, *P* = 0.034) decreased significantly after colonization, while the remaining phylum did not differ significantly (all *P* > 0.05).

At the genus level, the differences between the basal oral flora of groups E and C were not significant in the high-abundance genera (all *P* < 0.05), while there were some differences in the low-abundance genera (Table 1). After IOH patients’ salivary flora colonization in group E rats compared with group C in the same period, the proportions of *Streptococcus*, *Mycoplasma*, *Fusobacterium*, *Leptotrichia*, *Dietzia*, *Lachnoanaerobaculum*, *Haemophilus*, *Peptostreptococcus* and *Nitriliruptor* were significantly higher, while the proportions of *Facklamia*, *Cellulosimicrobium* and *Granulicatella* were significantly lower (Table 2). In group C, the salivary flora of IOH patients differentially changed in abundance in the top 50 genera after colonization compared to before colonization (Table 3). After salivary flora colonization of healthy subjects in Group E compared with before colonization, *Streptococcus*, *Aggregatibacter*, *Gemella*, *Facklamia*, *Staphylococcaceae_Staphylococcus*, *Enterococcus*, *Ralstonia*, *Mycoplasma* and *Sphingomonas* were significantly lower, while the proportions of *Rothia*, *Veillonella*, *Actinomyces graevenitzii*, *Neisseria*, *Bacillaceae_Bacillus* and *Actinobacillus* were significantly higher (Table 4). In addition, the proportions of *Fusobacterium*, *Leptotrichia*, and *Haemophilus* decreased after colonization compared to before colonization with healthy subjects’ salivary flora in Group E. However, the differences were not statistically significant (all *P* > 0.05) (Table 4). While no such manifestations were seen in group C.

**Table 1:** Baseline oral microflora difference between the two groups of rats (genus level)

Genus	Group E	Group C	Z-value	*P*-value
*Lactobacillus*	0.02125 ± 0.05720	0.03658 ± 0.07705	-2.205	0.027
*Staphylococcaceae_Staphylococcus*	0.00027 ± 0.00019	0.00390 ± 0.00502	-3.046	0.002
*Enterococcus*	0.00002 ± 0.00006	0.00082 ± 0.00076	-2.973	0.003
*Bifidobacterium*	0.00092 ± 0.00244	0.00086 ± 0.00067	-2.212	0.027
*Mycoplasma*	0	0.00116 ± 0.00194	-2.208	0.027
*Acinetobacter*	0.00005 ± 0.00010	0.00027 ± 0.00025	-2.412	0.016
*Erysipelotrichaceae_Clostridium*	0.00003 ± 0.00009	0.00059 ± 0.00085	-2.430	0.015
*Shigella*	0.00011 ± 0.00013	0.00050 ± 0.00027	-2.633	0.008
*Silene*	0	0.00010 ± 0.00017	-2.208	0.027
*Blautia*	0.00002 ± 0.00005	0.00099 ± 0.00237	-2.315	0.021
*Roseburia*	0	0.00085 ± 0.00171	-2.554	0.011

**Table 2:** The difference of oral flora between two groups of rats with IOH after salivary flora colonization (genus level)

Genus	Group E	Group C	Z-value	*P*-value
*Streptococcus*	0.480000 ± 0.070000	0.280000 ± 0.140000	-2.021	0.043
*Facklamia*	0.001000 ± 0.000300	0.020000 ± 0.020000	-2.309	0.021
*Mycoplasma*	0.002000 ± 0.002000	0.000090 ± 0.000100	-2.033	0.042
*Fusobacterium*	0.002000 ± 0.002000	0	-2.460	0.014
*Leptotrichia*	0.001000 ± 0.002000	0	-1.984	0.047
*Dietzia*	0.000200 ± 0.000100	0	-1.984	0.047
*Cellulosimicrobium*	0.000010 ± 0.000030	0.000200 ± 0.000040	-2.366	0.018
*Lachnoanaerobaculum*	0.000200 ± 0.000200	0	-1.984	0.047
*Granulicatella*	0.000003 ± 0.000006	0.000100 ± 0.000100	-2.366	0.018
*Haemophilus*	0.000100 ± 0.000060	0	-2.460	0.014
*Peptostreptococcus*	0.000080 ± 0.000050	0	-2.460	0.014
*Nitriliruptor*	0.000030 ± 0.000020	0.000020 ± 0.000020	-0.744	0.457

**Table 3:** Difference of salivary flora in Group C before and after colonization with IOH patients’ salivary flora (genus level)

Genus	Before-colonization	After-colonization	Z-value	*P*-value
*Rothia*	0.29538 ± 0.17628	0.04894 ± 0.05786	-2.208	0.027
*Aggregatibacter*	0.13611 ± 0.20731	0.51099 ± 0.25481	-2.038	0.042
*Variovorax*	0.07941 ± 0.08341	0.00034 ± 0.00047	-2.552	0.011
*Cellulosimicrobium*	0	0.00015 ± 0.00004	-3.233	0.001
*Bifidobacterium*	0.00871 ± 0.00903	0.00006 ± 0.00008	-2.552	0.011
*Ralstonia*	0.00002 ± 0.00006	0.00050 ± 0.00049	-2.651	0.008
*Ruminococcus*	0.00076 ± 0.00069	0.00003 ± 0.00005	-2.161	0.031
*Jeotgalicoccus*	0.00318 ± 0.00347	0.00003 ± 0.00007	-2.566	0.010
*Bacillaceae_Bacillus*	0.00137 ± 0.00169	0.00006 ± 0.00007	-2.378	0.017
*Acinetobacter*	0.00242 ± 0.00261	0.00021 ± 0.00040	-2.212	0.027
*Adlercreutzia*	0.00084 ± 0.00106	0.00006 ± 0.00011	-2.161	0.031
*Actinobacillus*	0	0.00073 ± 0.00133	-2.675	0.007
*Shigella*	0.00113 ± 0.00087	0.00003 ± 0.00006	-2.161	0.031
*Silene*	0.00148 ± 0.00211	0	-2.466	0.014
*Blautia*	0.00042 ± 0.00047	0	-2.176	0.030

**Table 4:** Difference of salivary microflora before and after colonization with healthy’ salivary flora in group E (genus level)

Genus	Before-colonization	After-colonization	Z-value	*P*-value
*Streptococcus*	0.47612 ± 0.06533	0.23152 ± 0.02637	-2.121	0.034
*Rothia*	0.05036 ± 0.03205	0.33565 ± 0.04539	-2.121	0.034
*Aggregatibacter*	0.29452 ± 0.06033	0.10290 ± 0.01860	-2.121	0.034
*Veillonella*	0.02203 ± 0.01989	0.11403 ± 0.04552	-2.121	0.034
*Gemella*	0.09711 ± 0.04063	0.01339 ± 0.00592	-2.121	0.034
*Actinomyces*	0.00604 ± 0.00353	0.04823 ± 0.01007	-2.121	0.034
*Facklamia*	0.00060 ± 0.00029	0.00419 ± 0.00286	-2.121	0.034
*Staphylococcaceae_Staphylococcus*	0.00131 ± 0.00090	0.00004 ± 0.00004	-2.121	0.034
*Enterococcus*	0.00494 ± 0.00265	0.00037 ± 0.00007	-2.121	0.034
*Ralstonia*	0.00145 ± 0.00073	0	-2.201	0.028
*Neisseria*	0.00002 ± 0.00003	0.00172 ± 0.00077	-2.201	0.028
*Mycoplasma*	0.00206 ± 0.00181	0.00007 ± 0.00006	-2.121	0.034
*Bacillaceae_Bacillus*	0.00007 ± 0.00003	0.00167 ± 0.00075	-2.121	0.034
*Actinobacillus*	0.00003 ± 0.00003	0.00141 ± 0.00075	-2.141	0.032
*Sphingomonas* *Fusobacterium* *Leptotrichia* *Haemophilus*	0.00020 ± 0.00009 0.00179 ± 0.00198 0.00133 ± 0.00217 0.00013 ± 0.00006	0 0.00097 ± 0.00045 0 0.00012 ± 0.00005	-2.201 0 -1.755 -0.354	0.028 1.000 0.079 0.724

### PICRUSt (phylogenetic investigation of communities by Reconstruction of Unobserved States) functional predictive analysis

Since the main substances that produce odor are VSCs, the metabolism of sulfur-containing amino acids is of particular interest. Compared to group C, group E rats with IOH exhibited significantly higher levels of cysteine and methionine metabolism, and arginine and proline metabolism after salivary flora colonization (Table 5), whereas this metabolic difference was not demonstrated at baseline (Table 6). However, cysteine and methionine metabolism, and arginine and proline metabolism did not show significant decreases in healthy subjects in Group E rats after salivary flora colonization compared to Group C (Table 7). However, cysteine and methionine metabolism decreased significantly, and arginine and proline metabolism also showed a decreasing trend after colonization compared to healthy subjects in Group E before salivary flora colonization (Table 8).

**Table 5:** Different metabolic pathways of oral flora in two groups of rats with IOH after salivary flora colonization

Metabolic pathways	Group E	Group C	Z-value	*P*-value
Cysteine and methionine metabolism	537.275 ± 17.397	457.321 ± 44.806	-2.309	0.021
Arginine and proline metabolism	248.201 ± 4.950	229.206 ± 11.810	-2.309	0.021

**Table 6:** Difference of initial IOH-related metabolic pathway between two groups of rats

Metabolic pathways	Group E	Group C	Z-value	*P*-value
Cysteine and methionine metabolism	470.45 ± 23.52	478.10 ± 28.31	-0.289	0.773
Arginine and proline metabolism	227.82 ± 19.83	232.37 ± 35.42	-0.289	0.773

**Table 7:** Metabolic pathway of oral flora after salivary flora colonization with healthy subjects in two groups of rats

Metabolic pathways	Group E	Group C	Z-value	*P*-value
Cysteine and methionine metabolism	488.516 ± 11.563	470.882 ± 8.897	-1.528	0.127
Arginine and proline metabolism	246.465 ± 7.943	242.102 ± 3.626	-0.655	0.513

**Table 8:** Metabolic pathways of oral flora in healthy subjects of group E rats before and after salivary flora colonization

Metabolic pathways	before-colonization	after-colonization	Z-value	*P*-value
Cysteine and methionine metabolism	537.275 ± 17.397	488.516 ± 11.563	-2.121	0.034
Arginine and proline metabolism	248.201 ± 4.950	246.465 ± 7.943	-0.354	0.724

### Changes in breath values in rats

**Fig. 5:**
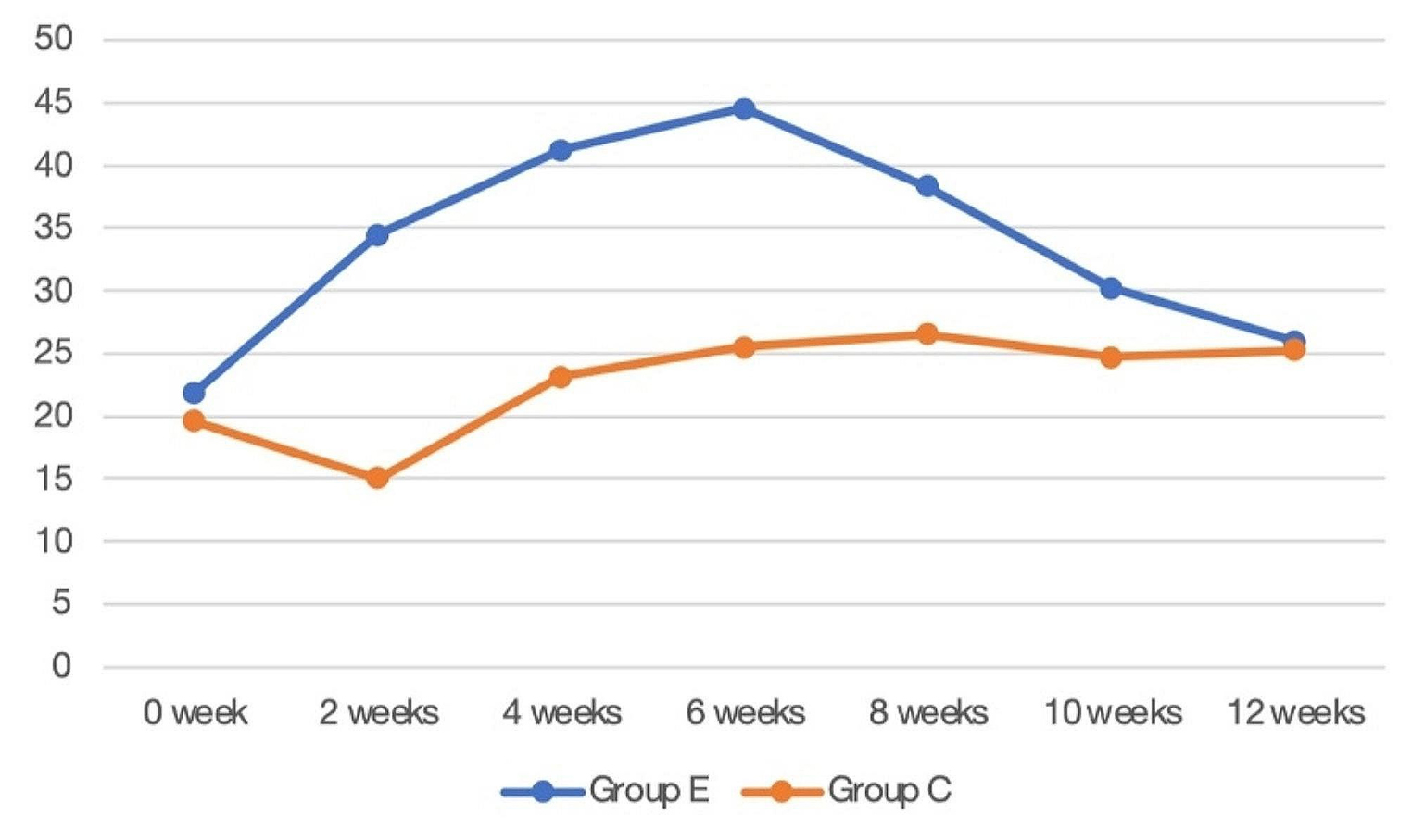
Folding line graph of changes in breath values of two groups of rats. Each point in the graph indicates the mean breath value of the two groups of rats at the same time

To understand the changes in breath values during the course of the oral cavity of rats in IOH patients and healthy subjects with salivary flora colonization of group E, breath tests were performed every 2 weeks in rats. There was a statistical difference in breath values after salivary flora colonization of IOH patients in group C compared to baseline (t=-2.250, *P* = 0.041), while there was a significant difference in group E (t=-9.144, *P* < 0.0001). And the difference in breath values after salivary flora colonization was significant between the two groups of IOH patients (t = 7.827, *P* < 0.0001). There was a significant difference in breath values after salivary flora colonization in healthy subjects in group E compared to IOH patients (t=-6.575, *P* < 0.0001), while the difference was not significant in group C (t = 0.128, *P* = 0.902). The difference in breath values between the two groups of healthy subjects after salivary flora colonization was not significant (t = 0.415, *P* = 0.689). The folded graph of the change in breath values of the rats in both groups is shown in Fig. 5.

## Discussion

### Halitosis and oral microecology

Halitosis can be divided into three categories, namely IOH, extra-oral halitosis (EOH), and transient halitosis, with IOH having a close relationship with oral microecology, EOH is mainly caused by systemic diseases (liver, kidney, and endocrine diseases, etc.) and certain medications [25, 26], while transient halitosis is caused by some foods (garlic and onion, etc.) or their residues cause. Oral microecology, as the second most complex microecological system after intestinal microecology, has numerous colonizing ecological sites, which Nicola Segata et al. [10] grouped into three groups according to the ratio of *Firmicutes* and *Bacteroidetes*: (i) oral mucosa, keratinized gingiva and hard palate; (ii) saliva and tongue; and (iii) supragingival and subgingival plaque. Studies of many oral diseases have found microecological differences detected in saliva and tongue for halitosis [27, 28], supragingival plaque for dental caries [29], and subgingival plaque for periodontitis [30], suggesting that oral flora plays an important role in the development of these diseases. Saliva represents the overall oral flora and it has been increasingly used as a source of DNA for epidemiological and population genetic studies [31], and today, when geography heavily influences the genetic structure, the salivary microbiome does not show great differences between geographic locations [32], and it also plays an important role in maintaining oral flora homeostasis [27]. Its main flora components are produced by its flushing of each ecological site of the oral cavity, and it is a common sample for studying oral flora because of its simple acquisition, high genetic stability, and representativeness [33, 34]. Therefore, in this study, salivary flora from IOH patients and healthy subjects were selected for the Wistar rat oral flora colonization test to further analyze the feasibility of OMT for IOH.

## Breath testing

Currently, the main equipment and methods commonly used for breath testing in clinical and research are the Halimeter breath detector, gas chromatography, and organoleptic scoring. Organoleptic scoring (OLS), which determines the presence and severity of halitosis by assigning a score, is widely used in halitosis research because it is easy to implement, but has the disadvantage of being highly subjective and lacking reproducibility [35]. The Halimeter breath detector determines bad breath by detecting the amount of VSCs in the gas, which is more objective than the organoleptic scoring method, but does not provide a differentiated display of the main substances that make up VSCs (hydrogen sulfide, methyl mercaptan, and dimethyl sulfide) and cannot detect other odor-causing substances (short-chain fatty acids such as propionic acid, butyric acid, cadaverine, putrescine, fecal odorant and indole [36]) [37]. Gas chromatography is considered the best way to detect not only VSCs, but also to quantitatively distinguish and precisely analyze the main odor-causing substances, and to detect other odor-causing substances, but it is rarely used in clinical practice because of its poor operability [38]. Therefore, combined with the fact that the main substances causing IOH are VSCs, a portable Halimeter breath detector was used in this study for IOH patient collection and rat breath testing.

### New strategy for microbiota transplantation

Microbiota transplantation (MT) is the process of taking microbiota from a specific site in a healthy subject and colonizing it in a specific way in the patient’s pathogenic site to re-establish a healthy micro-ecology for therapeutic purposes. This technique dates back thousands of years [39], when the Chinese herbalist Ge Hong used fecal suspensions from healthy subjects to treat patients with food poisoning and severe diarrhea, producing such good results that it came to be known as fecal microbiota transplantation (FMT). FMT is now a well-established treatment modality, first documented in patients with recurrent refractory Clostridium difficile infection (CDI) [40], while today the effectiveness of treatment for a variety of conditions such as inflammatory bowel disease, chronic transmission constipation, non-alcoholic fatty liver disease (NAFLD), and allergies have been reported from time to time [41, 42]. In recent years, studies related to flora transplantation techniques for the treatment of flora imbalance diseases have been rapidly conducted, including skin microbiota transplantation (SMT) for atopic dermatitis [43, 44], vaginal microbiota transplantation (VMT) for bacterial vaginitis [45, 46], oral microbiota transplantation (OMT) for periodontitis and dental caries [47, 48], and uterine microbiota transplantation (UMT) for endometritis [49]. However, the intestine, vagina, and uterus are closed environments with high flora stability and are not easily influenced by the external environment, while the oral cavity is an open environment and is easily influenced by diet and environment, thus the research development of OMT treatment is relatively slow. This study takes advantage of the significant differences in salivary flora between IOH patients and healthy subjects and their higher stability, and then proposes that OMT treatment with the aim of restoring oral flora microecology is a new strategy for the treatment of halitosis.

### Microbiota transplantation for intra-oral halitosis

In the Wister rat oral flora colonization test, the most representative salivary flora from IOH patients and healthy subjects were selected for the rat E group oral colonization test. 7 IOH patients’ salivary flora and 3 healthy subjects’ salivary flora were mixed before colonization to eliminate possible inter-individual differences. There was no significant difference in abundance and diversity between the two groups of rats before colonization, and Adonis analysis suggested that the differences between groups were small, and the differences in high abundance species at the phylum and genus level were not significant, all of which indicated good baseline consistency between the two groups of rats.

The diversity of rat flora in group E was significantly lower than that in group C after the colonization of salivary flora in IOH patients, indicating that the colonization of salivary flora in IOH patients reduced the diversity of oral flora, which may be related to the competitive effect between flora. However, Adonis analysis suggested that the differences between the two groups after colonization were still small, suggesting that the structure of the flora of group E after colonization was not significantly different from that of group C. However, the proportion of species associated with halitosis in IOH patients, such as *Fusobacteria*, *Porphyromonas*, *Fusobacterium*, *Gemella*, *Leptotrichia* [17], and *Peptostreptococcus*, was significantly higher after colonization of rats in group E, whereas the abundance of all these species was zero before colonization. In contrast, the abundance of *Fusobacterium*, *Leptotrichia*, and *Peptostreptococcus* remained zero and the abundance of *Porphyromonas* did not change significantly after colonization in group C, suggesting that salivary odor-causing bacteria in IOH patients could colonize in group E. No significant correlation with environmental factors was found. The data obtained from the Halimeter breath detector showed an increasing trend in both groups, but the salivary flora of IOH patients in group E showed a significant increase after colonization compared to baseline and a highly significant difference compared to group C after colonization, suggesting that environmental factors may have some influence on breath but the underlying cause is an imbalance of oral flora. PICRUSt metabolic prediction analysis showed that cysteine and methionine metabolic pathways and arginine and proline metabolic pathways were significantly enhanced in group E compared to group C after colonization, with statistically significant differences, while there was no such performance at baseline, and there was no statistically significant relationship between the target metabolic pathways at baseline and after colonization in group C. This suggests that halitosis-associated flora can clearly colonize and perform their metabolic functions, and the relationship with environmental factors remains insignificant. Combined with the above showed that the salivary flora of IOH patients in Group E had similar characteristics to those of clinical patients after colonization. The proportion of Fusobacterium, *Leptotrichia*, and *Haemophilus* that were clearly associated with IOH decreased after colonization compared to healthy subjects in Group E before salivary flora colonization. Compared with the salivary flora colonization of healthy subjects in group C, the flora diversity in group E was significantly higher, suggesting that the salivary flora colonization of healthy subjects restored the oral flora diversity in group E rats. PICRUSt metabolic prediction analysis suggested that healthy subjects in Group E showed a decrease in cysteine and methionine metabolism, and arginine and proline metabolism after salivary colonization compared to before colonization. Similarly, compared to healthy subjects in Group E before salivary flora colonization, breath values decreased significantly after colonization and approached the level of Group C. Although the changes in species composition and metabolic changes in Group E rats after salivary flora colonization in healthy subjects may be affected by confounding factors such as sample size and uncertain environment, there is a trend of correlated changes and significant changes in breath values, which can still indicate to some extent that OMT is effective in treating IOH, but further validation is still needed in a long colonization trial with a large sample. There was no significant difference in salivary flora alpha diversity between group C baseline and IOH patients after colonization, while PCoA analysis showed a significant trend of separation. Combined with the structural changes of low abundance flora at the phylum and genus level and the significant decrease in salivary flora alpha diversity in healthy subjects before and after colonization, it suggests that environmental factors can change the structure and diversity of oral flora but not the flora abundance.

### Translational value of microbiota transplantation for the treatment of intra-oral halitosis

In this study, we took IOH as the target problem, drew on the successful experience of FMT therapy, and referred to the results of previous animal studies on MT therapy for periodontitis, and then explored the feasibility of OMT for the treatment of IOH. First, the rat model of IOH was created using the salivary flora of IOH patients to colonize the oral cavity of rats, which maximally preserved the microbial interactions and metabolic networks, and well simulated the clinical symptoms, microbes, and their metabolic changes of the patients, which provided a prerequisite for the evaluation of the OMT; secondly, salivary flora from healthy subjects was used to colonize the oral cavity of model rats to indirectly demonstrate that OMT is feasible for the treatment of IOH by evaluating the changes in the structure of oral flora, metabolic pathways, and breath values in the model rats. Overall, the methodology, which uses salivary flora from IOH patients and healthy subjects for animal experiments and employs a clinical evaluation system, has findings that are feasible for clinical translation and provide important clues for the future development of emerging microbial therapeutics for IOH, and for research and translation in the areas of microbial-metabolomics mechanisms and target-flora interactions.

### Shortcomings of this study

This study still has the following limitations: (i) only a simple clinical assessment of the periodontal condition of the subjects was performed, without a systematic assessment of the dental profession (plaque index, gingival index, bleeding on probing, etc.), (ii) the PICRUSt metabolic prediction analysis used in this study can only preliminarily predict the metabolic function of the flora and cannot accurately assess it; (iii) the 16SrRNA sequencing technology used in this study can only preliminarily verify whether there is colonization of the target flora, but its origin still cannot be accurately determined; (iv) this study only indirectly explores the feasibility of OMT treatment through the colonizability of salivary flora in IOH patients and salivary flora in oral healthy subjects, which lacks systematicity.

### Outlook

Based on the experimental results of this study, we can make the following perspectives: (i) accurate assessment of halitosis-related metabolism using metabolomic approaches after systematic assessment of oral indicators, and construction of a metabolic network based on species symbiosis for in-depth study of flora interactions; (ii) accurate assessment of target flora colonization and viability using microbial tracer technology, or analysis based on single nucleotide polypeptide characterization, and systematic assessment of the safety and efficacy of OMT for IOH is further research directions.

## Conclusions

In conclusion, OMT treatment modality with the goal of restoring oral microecological balance is highly feasible and a promising green treatment method, and has some clinical translational value, but the influence of environmental factors and individual differences needs to be considered.

### Method

#### Sampling population

Seven patients with IOH who attended the First Affiliated Hospital of Xinjiang Medical University from June 2017 to June 2022 were selected, including four males and three females; ages 31–46 years, mean age (40.0 ± 2.79) years; three healthy subjects who were examined at the Health Management Center of the First Affiliated Hospital of Xinjiang Medical University during the same period were selected, including one male and two females; ages 27–45 years old, with a mean age of (35.67 ± 2.79) years. The gingival color of the seven patients with IOH and the three healthy subjects was pink, no bleeding gingiva, gingival margins closely fitting the teeth, no swelling or inflammation of periodontal tissues, no pain or discomfort, no significant gingival pocket formation or depth, no loosening of the teeth, and no significant differences in visual periodontal health, except for differences in breath. Inclusion criteria for IOH patients: (1) patients presenting to the First Affiliated Hospital of Xinjiang Medical University with complaints of halitosis; (2) periodontal health; (3) no systemic disease; (4) age ≥ 18 years; (5) breath test value ≥ 110 ppb at the time of consultation; (6) all included patients signed an informed consent form. Exclusion criteria for IOH patients: (1) those who have received periodontal treatment within 1 year; (2) those who have received topical or systemic antibiotics within 6 months; (3) those who have used non-steroidal anti-inflammatory drugs within 3 months; (4) smokers; (5) those who have systemic diseases; (6) those who are pregnant or breastfeeding; (7) those who are unable or unwilling to participate in this clinical trial study.

### Laboratory animals

Sixteen 6- to 8-week-old SPF-grade Wistar rats with an average weight of 254.27 ± 37.06 g, male, in general health, with production license SCXK (Xin) 2018-0003, were selected from the Animal Experiment Center of Xinjiang Medical University. The rats were kept in the following environments: (i) barrier environment: temperature 23°∽25°, relative humidity 50%∽70%, alternating light and darkness; (ii) general environment: temperature 18°∽26°, alternating day and night for 12 h. Barrier environment rearing for sterile diet and water, and general environment rearing for specific pathogen-free diet and water. All rats changed their bedding every 5 days, and salivary colonies of IOH patients were not restricted in diet and water before colonization. After colonization, group E was continuously given sucrose water at a concentration of 10%. At the end of the experiment, we placed the Wistar rats into a CO_2_ execution chamber and euthanized the rats by CO_2_ inhalation. This method can be used to euthanize multiple rats at the same time without destroying the body structure of the rats, which is safer and more reliable than other methods. The animal experimental procedures all conformed to the ethical requirements of animal experiments and were approved by the Experimental Animal Ethics Committee of Xinjiang Medical University (approval number: IACUC-20210405-14).

### Sample collection and experimental procedure

Saliva sample collection: 2.5 ml of non-stimulating saliva (saliva naturally secreted by the salivary glands in the absence of any stimulation) was collected in the morning without oral cleaning and under fasting conditions, placed in lyophilization tubes, all immediately snap frozen in liquid nitrogen, and then transferred to -80℃ refrigerator for freezing and storage after all samples were collected. Collection of oral flora samples from rats: Before each sampling, anesthesia was induced by using isoflurane (flow rate of 4 L/min, concentration of 4%) for 1 min, then the rats were fixed on the test bench, and the teeth and oral mucosa of the rats were wiped with disposable sterile swabs for 3 times, then put into lyophilized tubes with PBS buffer, and then put into the freezer for freezing at -80 °C.

Configure 2% sodium carboxymethyl cellulose, 0.1% sodium hypochlorite solution, and 23µM sodium ascorbate, the above reagents are ready to use. Salivary flora of 7 IOH patients and 3 healthy subjects were mixed separately, and 2% sodium carboxymethylcellulose was added in equal volume, and then PBS containing 20% glycerol was added in equal volume, dispensed (2 ml per unit) and stored in -80 °C refrigerator.

**Fig. 6:**
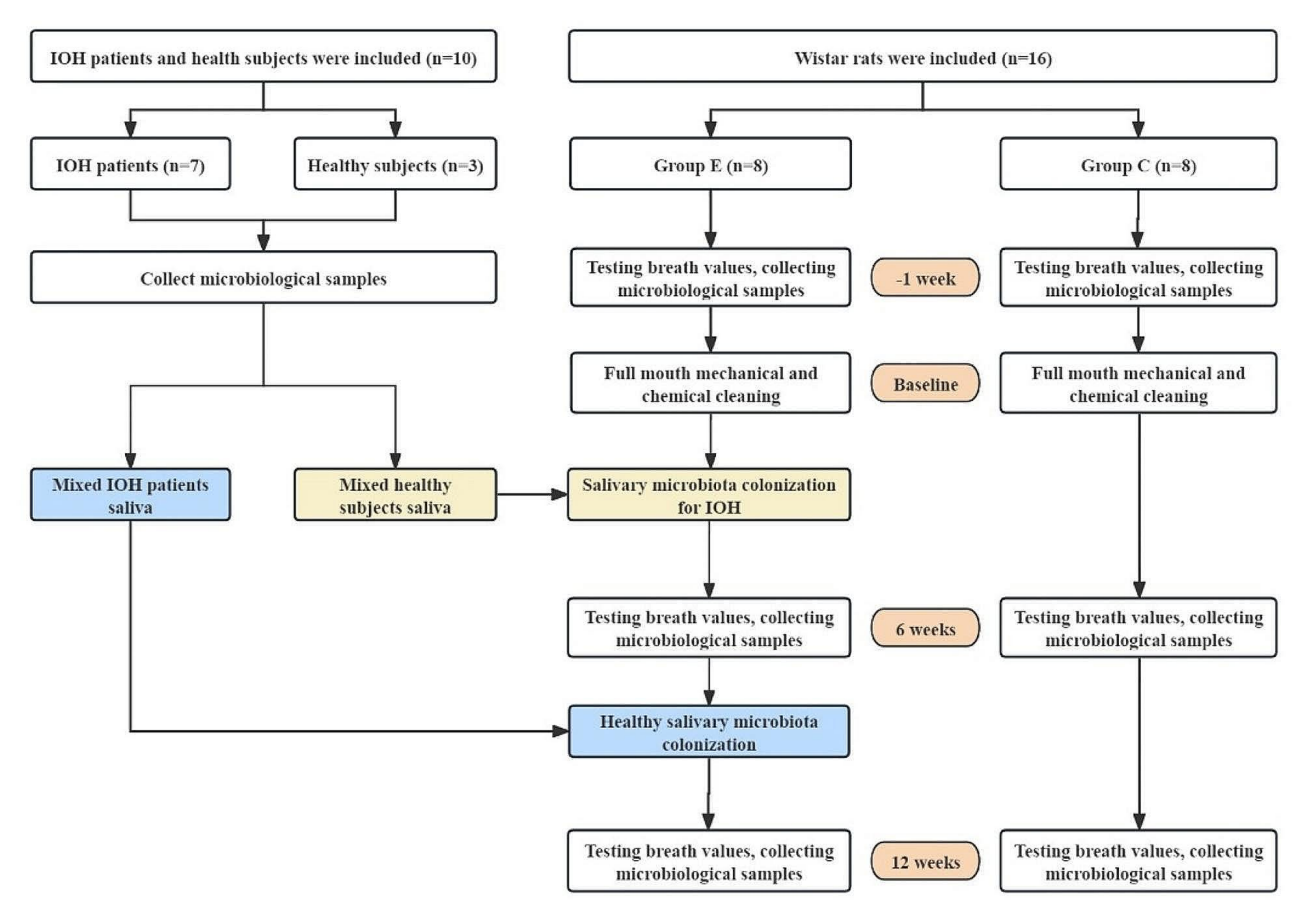
Framework diagram of the study structure

Experimental procedure: The structural framework of the study is illustrated in Fig. 6. (1) In the morning fasting state, the ppb value of gas in the mouth was detected using a portable Halimeter breath detector (specification model: RH-17 K, manufacturer: Interscan Corporation, USA), and the test was repeated three times with an interval of 5 min each time, and the average value was taken. According to the instructions for use (http://halimeter.com/ calibration-procedure), ppb ≥ 110 was defined as halitosis; ppb < 110 was defined as normal breath. Basic information of the included subjects is shown in Table 9. Saliva samples were retained from IOH patients and healthy subjects, respectively. (2) All rats were housed in the barrier for 1 week and then sampled under anesthesia for oral flora, randomly divided into group E and group C, 8 rats in each group, all marked with ear tags and detected the gas ppb value in the oral cavity of the rats using a portable Halimeter breath detector, repeated 3 times with an interval of 5 min each time, and the average value was taken, and the basic information of the experimental rats is shown in Table 10. Then all the teeth and mucous membranes of the whole mouth were cleaned mechanically using a swab dipped in saline after anesthesia in the general environment. After 1 week of routine feeding, the whole mouth was rinsed with 0.1% NaoCl for 5 min after repeated cleaning of the whole teeth and mucous membranes, then rinsed again with 23 µM buffered sodium ascorbate for 10 min for inactivation, and again under anesthesia for sampling of oral flora [48]. After using the dispensed IOH patient bacterial solution to apply to the teeth and oral mucosa of group E rats (how to apply: use a 1 ml syringe with the needle removed to apply slowly in small portions), each rat was given 0.25 ml once a day, with water fasting for 1 h after application, followed by continuous administration of 10% sucrose water for 6 weeks after oral flora sampling under anesthesia. Group C was routinely reared and sampled during the same period. Group E continued to give bacteriophage transplants to healthy subjects for 6 weeks and sampled under the above conditions in a general environment. Group C was routinely reared and sampled during the same period. The experimental procedure was performed every 2 weeks using a Halimeter breath detector under anesthesia to measure the ppb values of the gases in the mouth of both groups.

**Table 9:** Basic information of the included study subjects

Clinical Indicators		Group H (*n* = 7)	Group N (*n* = 3)	t/χ^2^	*P*-value
Age (years)		40.00 ± 2.79	35.67 ± 2.79	1.088	0.308^*^
Gender	Female	3(43.0%)	2(67.0%)	0.218	0.490^$^
	Male	4(57.0%)	1(33.0%)		
Breath value (ppb)		211 ± 93	65 ± 18	2.610	0.031^*^

* denotes calculation using two independent samples t-test and $ denotes calculation using chi-square test

**Table 10:** Basic information for inclusion in Wistar rats

Testing index	Group E (*n* = 8)	Group C (*n* = 8)	t	*P*-value
Breath value (ppb)	22 ± 5	20 ± 6	0.847	0.411^*^
Body weight (g)	256.7 ± 27.9	251.9 ± 46.4	0.251	0.805^*^

* indicates calculation using two independent samples t-test

### Experimental reagents

Sodium carboxymethyl cellulose (25 g) and sodium ascorbate (100 g) were purchased from Beijing Solabao Technology Co. The item numbers are C8621 and S9440, respectively. Sodium hypochlorite solution (500 ml) and glycerol (500 ml) were purchased from Shanghai Maclean Biochemical Technology Co. Item number S817441 and G6201 respectively. PBS (500 ml) was purchased from Beijing Xino Factor Technology Co. Item number is CBS004S-BR500. Isoflurane (100 ml) was purchased from Hebei Jindafu Pharmaceutical Co. Lot number 20,220,202. Sodium chloride injection (500 ml) was purchased from Sichuan Kellen Pharmaceutical Co. Lot number L122041203.

### DNA extraction and PCR amplification

DNA extraction, quality control, amplification, and purification were all done by Shanghai Personal Biotechnology Co., Ltd. The amplified region was the conserved region of bacterial 16 S rRNA V3-V4 with primers F: ACTCCTACGGGAGGCAGCA R: GGACTACHVGGGTWTCTAAT. The PCR amplification steps were: pre-denaturation at 98 °C for 2 min; denaturation at 98 °C for 15s; annealing at 55 °C for the 30s; extension at 72 °C for 30s, for a total of 25–30 cycles; and final extension at 72 °C for 5 min. The amplified DNA was double-end sequenced by the Novaseq-PE250 sequencing platform (produced by Illumina, USA) for DNA fragmentation and subsequent bioinformatics analysis.

### Data organization and bioinformatics analysis

All raw data were recorded using Excel spreadsheets. The QIIME2 dada2 analysis process was chosen to denoise the sequences, and the resulting several denoised sequence ASVs were clustered at 100% similarity. The Silva database [50] was used and the classify-sklearn algorithm of QIIME2 [51], for each ASVs feature sequence, was used in the QIIME2 software with default parameters, using a pre-trained Naive Bayes classifier into species annotation. The Chao1 index and Observed species index were used to assess the abundance of the samples, and the Shannon index and Simpson index were used to assess the diversity of the samples. Principal coordinates analysis (PCoA) was generated using QIIME2 software, based on Bray-Curtis distance.

### Statistical methods

Data were processed using SPSS 26.0 software, and the mean ± standard deviation was used to describe the measurement data conforming to the normal distribution, and the t-test was preferred for categorical data. The QIIME2 platform was used to analyze the species composition, Alpha and Beta diversity of different sample data separately. Adonis analysis was used to test the significance of differences between groups, and the rank sum test (Mann-Whitney U test) was used for comparison between groups. The test levels were all α = 0.05.

## Data Availability

The raw sequence data reported in this paper have been deposited in the Genome Sequence Archive (Genomics, Proteomics & Bioinformatics 2021) in National Genomics Data Center (Nucleic Acids Res 2022), China National Center for Bioinformation / Beijing Institute of Genomics, Chinese Academy of Sciences (GSA: CRA016060) that are publicly accessible at https://bigd.big.ac.cn/gsa/browse/CRA016060.

## Abbreviations

IOH: Intra-oral halitosis
VSC: Volatile sulfur compounds
EOH: Extra-oral halitosis
OLS: Organoleptic scoring
PICRUSt: Phylogenetic Investigation of Communities by Reconstruction of Unobserved States
FMT: Fecal Microbiota Transplantation
SMT: Skin Microbiota Transplantation
VMT: Vaginal Microbiota Transplantation
OMT: Oral Microbiota Transplantation
UMT: Uterine Microbiota Transplantation
ASV: Amplicon sequence variants
PCoA: Principal coordinates analysis
MT: Microbiota transplantation
CDI: Clostridium difficile infection
NAFLD: Non-alcoholic fatty liver disease
